# Nitrous Oxide as an Anæsthetic

**Published:** 1877-06

**Authors:** H. Gibbons


### ARTICLE IV.

### *A Personal Experience of Nitrous Oxide as an Anæsthetic.*

BY H. GIBBONS, M. D.

The experience of disease and of the action of medicines on one's own person often brings to light facts which could not possibly be learned through observations made on others. I have often thought that the subjective phase of medical enquiry is too much neglected, and that we depend too exclusively in our investigations on objective enquiry. A

good, round fit of sickness, with the consequent treatment, experienced by a medical student, will give him a quicker insight into the nature of the disease than he can gather from books or patients. Every medical student, both before and after commencing practice, but more particularly after, should make the best of all aberrations from health occurring in his own person. He should observe every thing with microscopic eye. He should subject every thing to mental analysis. He should record and remember everything.

A few months ago I had some teeth extracted whilst under the influence of nitrous oxide gas. Some new experiences derived from the process I propose to narrate, even at the risk of being charged with wasting time on a trivial subject. I was in Philadelphia, and suffered from my teeth for several weeks, but being unnerved by some months of invalidism, and having from early life endured much pain from my teeth and from the extraction of them, which was always difficult, I bore the pain in the hope of spontaneous relief. Disappointed in this I finally surrendered, and called on a brother residing in Philadelphia for reference to some good dentist. It was not my design to use an anæsthetic. My brother told me of a Dr. Thomas, who made a specialty of tooth-drawing under the influence of nitrous oxide gas, and who had extracted a larger number of teeth than any other living man—as was supposed. Dr. Thomas had lately extracted a tooth for my brother, who gave me a very intelligent and interesting account of his sensations on the occasion. The operator advised him to fix his thoughts on some subject whilst inhaling the gas. He chose mentally the subject of death, and passed into a sort of dream, imagining himself crossing a long bridge with other travelers, who were continually dropping through one by one. It was in fact a resurrection in his memory of the vision of Addison, one of his school boy readings. After he had been marching onward for a considerable time, expecting to fall through like his companions, he heard a sharp tinkling sound, and in the next moment felt a tap on the shoulder and heard the voice of the operator calling him by name. He then aroused instantly and completely, and was amazed to find his tooth lying by his side on the table. He was so enthusiastic in his account of the process and so charmed with the result, that a listener might almost feel tempted to have his teeth drawn in the same style for the mere luxury of it.

I repaired forthwith to the office of Dr. Thomas, in Walnut street, introduced myself, and took my seat in the official chair. There were three teeth, or rather the remains of three, causing the trouble, one canine and two adjoining in the lower jaw. The canine was the principal offender and I intended to have it alone extracted, and not to use an anæsthetic, but simply to avail myself of the skill of the operator. But before I realized my position the breathing tube was in my mouth and the gas was under respiration. I expected to feel its exhilarating effects, which I had many times experienced when inhaling it for amusement. But no such effect was produced; or if there were, no recollection of it remained. On the contrary the loss of consciousness was sudden and complete. There was no dreaming nor any sense of the lapse of time such as my brother had experienced. But a sharp tinkling sound, barely audible, was the only sensation of any kind. In the next moment, I felt a gentle tap on the hand and heard the voice of the operator, and returned in a flash as it were, to perfect consciousness. I did not realize the lapse of an iota of time from the inhalation of the gas till the restoration of consciousness, and of course was much surprised when Dr. Thomas pointed to the table at my side, saying "There are your three teeth, Doctor!"

A slight degree of languor which followed, made it comfortable to me to keep my seat for about ten minutes. After that time no abnormal sensations were experienced. Not a trace of suffering remained in the mouth or followed the operation, nor did there follow the shadow of disturbance of the head or stomach.

Both in my brother's case and my own the tinkling sound appears to have been caused by the act of laying down the forceps on the marble slab after the extraction of the teeth. The dreaming in the one case, indicated that the entire brain was not anæsthetized, and that the condition in this respect approached that of normal sleep. But in my case the complete annihilation of time from the sharp line of departing consciousness to the equally sharp line of its perfect restoration, was the most striking idea furnished by the entire proceeding.

There is probably nothing peculiar in all these details—nothing more than has been experienced by thousands of persons. Judging from the descriptions given me by others, I presume there is much sameness in the sensations resulting from the same causes. Patients inform me that they very generally awake from anæsthesia with the most confident impression that there has not been time to do any thing, and that their revival is premature; and that they are amazed and perplexed to find their teeth on the table. I believe the idea of complete annihilation of time is much more marked in anæsthesia from nitrous oxide gas than from chloroform and ether.

In comparing the physiological action of the several agents, Dr. Thomas thinks that the gas is safer than others because its action is confined to the cerebrum and to the sensory tract. Never paralyzing the cerebellum or medulla oblongata, it does not disturb the function of respiration nor the action of the heart. In the many thousands of patients on whom he has operated under its influence, he has never met with a single instance of dangerous results. It is the practice in Philadelphia for operating dentists to send their patients to a specialist for the extraction of teeth—most of them having abandoned this branch of the business, in order that their patients may avail themselves of the benefits of the gas. Where a few teeth only are to be taken out from time to time they find it expensive to keep always on hand fresh supplies of nitrous oxide. Dr. Thomas appears to be the general favorite, to whom most of the patients are sent. Confining himself entirely to this special department and performing no other dental operations, there is probably no individual in the world superior to him in the art of tooth drawing. In all the Atlantic cities nitrous oxide is preferred to all other anæsthetics in painful dental operations, requiring but a brief time for their performance. In fact, it ought to supercede all other anæsthetic agents. The danger from it is simply *nil.* It is true that a few mishaps have been reported as the result of its prolonged use in surgical operations. But I cannot learn that a single death has ever occurred from it in a dentist's chair ; and the number of cases in which it has been employed in this way amounts to millions. Chloroform has a very different record, and though ether is nearly exempt from suspicion of fatal effects, yet the nausea which almost invariably follows its inhalation presents a strong argument against it, which is not in the least degree applicable to the gas. Besides, the inhalation of the gas is easily accompilished and is not disagreeable. Chloroform is unpleasant to patients generally, and ether much more so. The anæsthetic action of the gas is complete in one minute or less, whilst the other agents require an indefinite time for their operation.

In all brief surgical operations calling for anæsthesia, the same arguments apply in favor of nitrous oxide as in tooth drawing. But as the loss of sensation lasts only for one or two minutes, it is necessary to repeat the inhalation when the operations require more time. Patients have in this way frequently been kept under its influence for fifteen or twenty minutes. Such prolongation however of the anæsthetic state involves some risk and is not deemed advisable.

There is no question but that nitrous oxide would by this time have completely excluded other anæsthetics from dentistry, were it not for the difficulty before referred to, in keeping a supply of the article in a perfect condition. To this may be added as an objection the difficulty of transpor tation. For the purpose of transportation, tin vessels have been devised, in which the gas may be compressed. It has even been compressed, in some instances, into the liquid form.

Every one has observed, during surgical operations on anæsthetized subjects, the frequent struggles and contortions of the patient, indicate suffering. At least this condition exists when the agent, whether chloroform or ether, has not pushed to its extreme effect. In such cases it is difficult to avoid the impression that the patient really suffers pain; although on awaking he has no remembrance of it. The action of nitrous oxid is nearly exempt from these expressions of pain, and if they are really what they seem to be, this is an argument in its favor.

The leading points which I have endeavored to set forth in this paper are as follows:

Nitrous oxide gas is preferable to ether and chloroform as an anæsthetic, because,

1. It is much more easily administered, and is not unpleasant to the patient.

2. It is more speedy and certain in its action, exhibiting its effect in a definite time.

3. Its anæsthesia is as perfect as that from ether and chloroform, if not more so.

4. It has no disagreeable after effects.

5. It is absolutely safe, limiting its paralyzing power to the cerebrum and sensory tract, and not invading the cerebellum or medulla oblongata.

6. It is adapted to all brief surgical operations.

7. Its anæsthetic effect may be prolonged by renewal of inhalation, say for five or ten minutes, but should not be prolonged indefinitely. It is therefore not adapted for operations requiring a long time, such as ovariotomy.—*Pacific Med. and Surg. Journal.*

